# Data on changes in red wine phenolic compounds, headspace aroma compounds and sensory profile after treatment of red wines with activated carbons with different physicochemical characteristics

**DOI:** 10.1016/j.dib.2017.03.055

**Published:** 2017-04-08

**Authors:** Luís Filipe-Ribeiro, Juliana Milheiro, Carlos C. Matos, Fernanda Cosme, Fernando M. Nunes

**Affiliations:** a CQ-VR, Chemistry Research Centre, University of Trás-os-Montes and Alto Douro, School of Life Sciences and Environment, Chemistry Department, 5000-801 Vila Real, Portugal; b CQ-VR, Chemistry Research Centre, University of Trás-os-Montes and Alto Douro, School of Life Sciences and Environment, Biology and Environment Department, Edifício de Enologia, 5000-801 Vila Real, Portugal

**Keywords:** Red wine, 4-ethylphenol, 4-ethylguaiacol, Activated carbon, Chromatic characteristics, Phenolic compounds, Headspace aroma, Sensory characteristics

## Abstract

Data in this article presents the changes on phenolic compounds, headspace aroma composition and sensory profile of a red wine spiked with 4-ethylphenol and 4-ethylguaiacol and treated with seven activated carbons with different physicochemical characteristics, namely surface area, micropore volume and mesopore volume (“Reduction of 4-ethylphenol and 4-ethylguaiacol in red wine by activated carbons with different physicochemical characteristics: impact on wine quality” Filipe-Ribeiro et al. (2017) [1]). Data on the physicochemical characteristics of the activated carbons are shown. Statistical data on the sensory expert panel consistency by General Procrustes Analysis is shown. Statistical data is also shown, which correlates the changes in chemical composition of red wines with the physicochemical characteristics of activated carbons used.

**Specifications Table**

**Table:** 

Subject area	*Chemistry*
More specific subject area	*Food and Wine Chemistry*
Type of data	*Table, graph, figure*
How data was acquired	*Quantachrome (Nova 4200e)*
	*FTIR (Unicam Research Series)*
	*HPLC (Ultimate 3000, Dionex) with a Photodiode array detector (PDA-100, Dionex)*
	*GC–MS (Thermo-Finningam) with CombiPAL automated HS-SPME (CTCANALYTICS, AG)*
Data format	*Analysed*
Experimental factors	*Wine sample was spiked with two levels of 4-ethylphenol (1500* *μg/L and 750* *μg/L) and 4-ethylguaicol (300* *μg/L and 150* *μg/L) and treated with seven activated carbons with different physicochemical characteristics.*
Experimental features	*Activated carbons adsorption isotherms were analysed by gas adsorption and mercury porosimetry, surface groups were analysed by FTIR.*
	*Wine phenolic acids and anthocyanins were analysed by RP-HPLC with a photodiode array detector and headspace aroma compounds were analysed by headspace solid phase microextraction using a 50/30* *μm DVB/Carboxen/PDMS fibre followed by GC–MS using an Optima FFAP column (30 m×0.32* *mm, 0.25* *μm). Sensory analysis was performed by an expert panel of six experts.*
Data source location	*Vila Real, Portugal*
Data accessibility	*Data with this article*

**Value of the data**

• Data from this research highlights the effect of the physicochemical characteristics of activated carbons on the phenolic, headspace aroma and sensory profile of wines spiked with 4-ethylphenol and 4-ethylguaiacol.
• We analysed the phenolic profile by RP-HPLC and the aroma compounds by HS-SPME-GC/MS in red wines treated with activated carbons presenting different physicochemical characteristics and the results were analysed by principal component analysis for highlighting relations between chemical composition of red wines and physicochemical characteristics of activated carbons.
• Activated carbons removal efficiency of red wine ethylphenols was related to their surface area and micropore volume.
• High surface area of mesopores and total pore volume were important for the anthocyanin removal and decrease in colour intensity.
• This data could serve as a benchmark for other researchers, evidencing the influence of activated carbons treatment on the individual phenolic, chromatic and aroma compounds and sensory profile of red wine.

## 1 Data

**Fig. 1:**
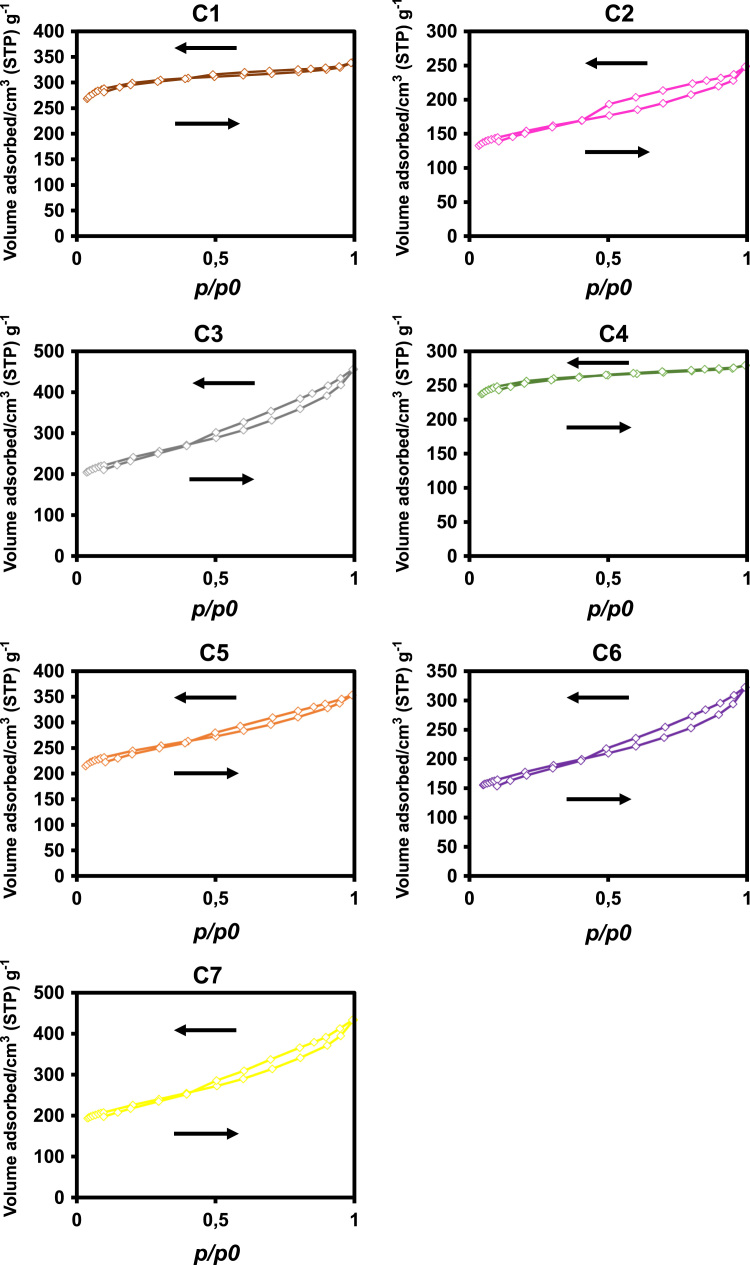
Adsorption isotherms (N_2_, −196 °C) of activated carbons; → adsorption; ← desorption.

**Fig. 2:**
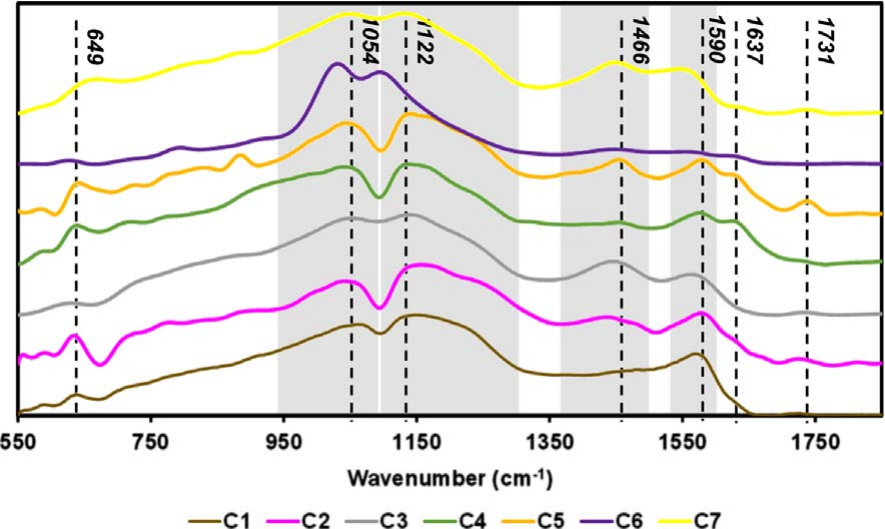
FTIR spectra of activated carbons.

**Fig. 3:**
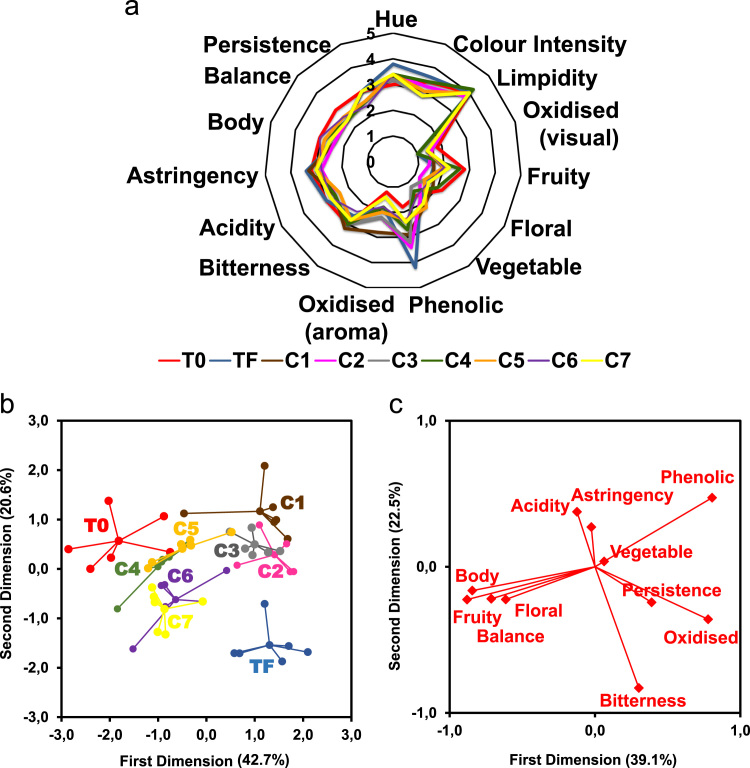
a) Sensory profile of volatile phenols free (T0) and volatile phenols spiked (TF) red wines and wines treated with the seven ACs (C1–C7); Consensus configuration for red wines treated with ACs with different physicochemical properties for removing 4-Ethylphenol and 4-Ethylguaiacol and sensory attributes; b) projection of wine samples and clouds for the first two dimensions and c) projection of sensory attributes on the first and second dimensions of Generalised Procrustes Analysis [5].

**Fig. 4:**
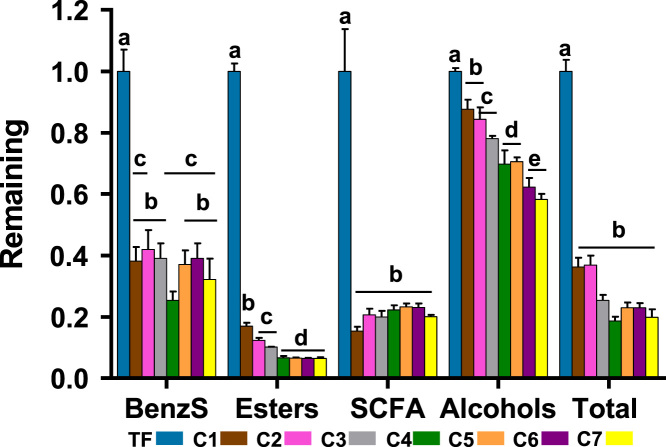
Reduction of total aroma compounds and of each class of chemical compounds after treatment with seven activated carbons, C1–C7 in relation to volatile phenols spiked (TF) red wines. BenzS – compounds containing a benzene in their structure. SCFA – short chain fatty acids. Error bars represent the standard deviation (n=4). Means followed by the same letter are not significantly different ANOVA and Tuckey post-hoc test (*p*<0.05).

**Fig. 5:**
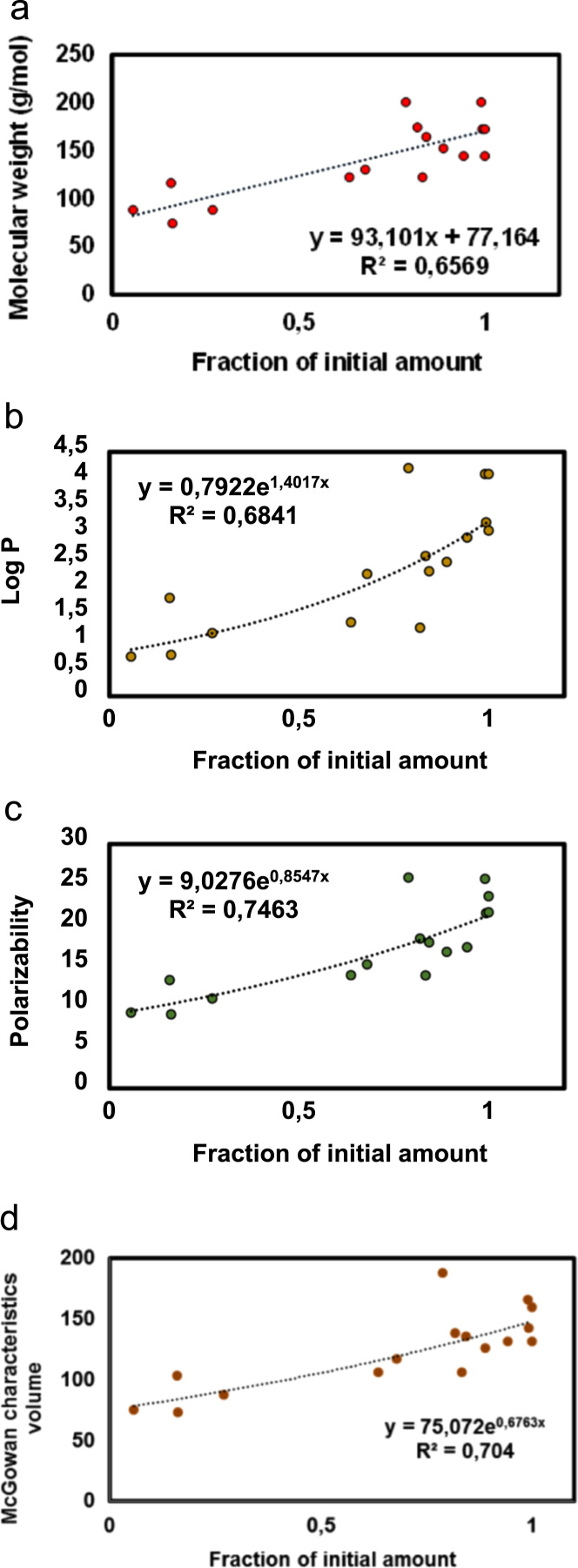
Correlation between fractions of headspace aroma average content of wines treated with activated carbons with a) molecular weight of aroma compounds; b) Log P of aroma compounds; c) polarizability of aroma compounds; d) McGowan characteristic volume.

**Fig. 6:**
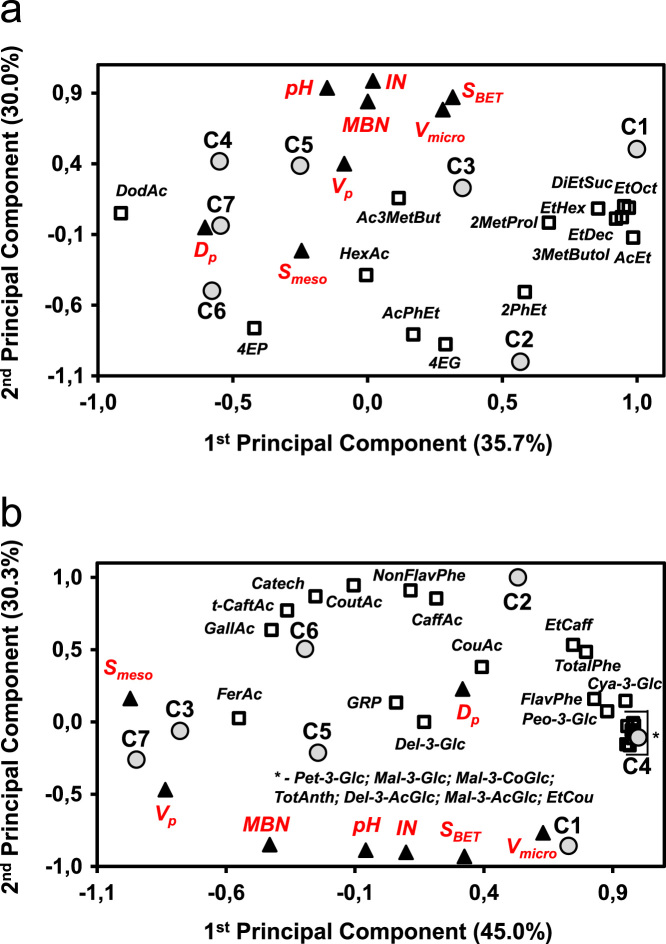
PCA that relate the AC characteristics with the: a) aromas and b) phenolic compounds. Red wines treated with seven ACs, C1 to C7; *S*_BET_-Brunauer-Emmett-Teller (BET) surface area; *S*_meso_-surface area of mesopores; *V*_*p*_-total volume of pores; *V*_micro_-micropore volume; *D*_p_-average pore diameter; IN–iodine adsorption number; MBN–methylene blue number; 2MetProl-2-Methylpropan-1-ol; Ac3MetBut-3-Methylbutan-1-ol acetate; 3-MetButol-3-Methylbutan-1-ol; EtHex-Ethyl hexanoate; EtOct-Ethyl octanoate; EtDec-Ethyl decanoate; DiEtSuc-Diethyl succinate; AcPh-Phenylethyl acetate; HexAc-Hexanoic acid; 2PhEt-2-Phenylethanol; 4-EG-4-Ethylguaiacol; 4-EP-4-Ethylphenol; DodAc-Dodecanoic acid. TotAnt–Total anthocyanins; TotPhe–Total phenols; FlavPhe–Flavonoid Phenols; NonFlavPhe–Non-Flavonoid Phenols; GallAc-Gallic acid; Catech–Catechin; t-CaftAc-*trans*-caftaric acid; GRP-2-*S*-glutathionyl caftaric acid; CoutAc-Coutaric acid; CaffAc-Caffeic acid; CouAc-Coumaric acid; FerAc-Ferulic acid; EtCaff-Caffeic acid ethyl ester; EtCou-Coumaric acid ethyl ester; Del-3-Glc-Delphinidin-3-glucoside, Cya-3-Glc-Cyanidin-3-glucoside, Pet-3-Glc-Petunidin-3-glucoside, Peo-3-Glc-Peonidin-3-glucoside, Mal-3-Glc-Malvidin-3-glucoside, Del-3-AcGlc-Delphinidin-3-acetylglucoside, Mal-3-AcGlc-Malvidin-3-acetylglucoside, Mal-3-CoGlc-Malvidin-3-coumaroylglucoside.

The data reported includes information about the adsorption isotherms of activated carbons (ACs) (Fig. 1), metal composition of activated carbons (Table 1) and surface group chemistry of activated carbons (Fig. 2 and Table 2). Also the sensory profile of wines (Fig. 3a) and consistency of the sensory panel scores were analysed by General Procrustes Analysis (GPA) (Fig. 3b and Table 3) and the scaling factor of each expert were determined (Table 4). The headspace aroma profile of red wines before and after treatment with activated carbons were determined (Table 5) and the reduction of total aroma compounds and reduction of each class of chemical compounds were calculated (Fig. 4). The headspace aroma compounds decrease and structural characteristics of each aroma compound were correlated (Table 6 and Fig. 5). The phenolic composition (total phenols, flavonoid phenols, non-flavonoid phenols, total anthocyanins) and colour properties (colour intensity, hue and chromatic characteristics) of treated and untreated wines were determined (Table 7). The phenolic profile of wines were determined by RP-HPLC that included the phenolic acids and flavonoids (Table 8) and monomeric anthocyanins (Table 9). The relation between aroma abundance and the activated carbons physicochemical characteristics were analysed by principal component analysis (Fig. 6a) and between the phenolic compounds content and activated carbons physicochemical characteristics (Fig. 6b).

## 2 Experimental design, materials and methods

### 2.1 Wine sample

A red wine from Douro Valley (vintage 2013) was used in this work, their main characteristics were follows: alcohol content 13.4% (v/v), specific gravity (20 °C) 0.9921 g/mL, titratable acidity 5.1 g/L expressed as tartaric acid, pH 3.84, volatile acidity 0.50 g/L expressed as acetic acid.

### 2.2 Analysis of conventional oenological parameters

Alcohol, specific gravity, pH, titratable acidity and volatile acidity were analysed using a FTIR Bacchus Micro (Microderm, France).

### 2.3 Experimental design

The addition of 4-ethylphenol and 4-ethylguaiacol was carried out on the red wine sample at the highest concentrations found in literature, 1500 μg/L for 4-ethylphenol and 300 μg/L for 4-ethylguaiacol (4-EP1500 and 4-EG300) [18] and were also prepared at medium level of contamination with 750 µg/L of 4-ethylphenol and 150 µg/L of 4-ethylguaiacol (4-EP750 and 4-EG150). Seven solid commercial activated carbons, characterized by [1], were used: C1 (powder), C2 (powder), C3 (powder), C4 (powder), C5 (powder), C6 (granulated) and C7 (powder). The activated carbons were next added at 100 (g/hL) maximum dosage authorized [19] to the wine placed in 250 mL graduated cylinders. After 6 days the wines were removed from graduated cylinders and then were centrifuged at 10,956*g*, 10 min at 20 °C in order to be analysed. All the assays and analyses were performed in duplicate.

### 2.4 Colour and total anthocyanins

Colour intensity and hue was determined by measuring absorbance at 420 nm, 520 nm and 620 nm (1 mm cell) according to [20]. The content of total anthocyanins was determined according to [21].

### 2.5 Chromatic characterization

The chromatic characteristics of wines calculated using the CIELab method according to [20]). The colour difference was calculated using the following equation: ΔE*=[(ΔL*)^2^+(Δa*)^2^+(Δb*)^2^]^1/2^.

### 2.6 Quantification of non-flavonoids, flavonoids and total phenols

The phenolic content of the wines was quantified using the absorbance at 280 nm before and after precipitation of the flavonoid phenols, through reaction with formaldehyde, according to [22]. The results were expressed as gallic acid equivalents by means of calibration curves with standard gallic acid. The total phenolic content was also determined by a spectrophotometric method, using a UV–vis spectrophotometer according to [23].

### 2.7 High performance liquid chromatography (HPLC) analysis of anthocyanins and phenolic acids

Analyses were carried out with an Ultimate 3000 HPLC equipped with a PDA-100 photodiode array detector and an Ultimate 3000 pump according to [24]. Quantification was performed with calibration curves with standards caffeic acid, coumaric acid, ferulic acid, gallic acid and catechin. The results of *trans*-caftaric acid, 2-*S*-glutathionylcaftaric acid (GRP) and caffeic acid ethyl ester were expressed as caffeic acid equivalents by means of calibration curves with standard caffeic acid. On the other hand, coutaric acid, coutaric acid isomer and coumaric acid ethyl ester were expressed as coumaric acid equivalents by means of calibration curves with standard coumaric acid. A calibration curve of malvidin-3-glucoside, cyanidin-3-glucoside and peonidin-3-glucoside were used for quantification of anthocyanins. Using the coefficient of molar absorptivity (ε) and by extrapolation, it was possible to obtain the slopes for delphinidin-3-glucoside, petunidin-3-glucoside, and malvidin-3-coumaroylglucoside and perform the quantification. The results of delphinidin-3-acetylglucoside, petunidin-3-acetylglucoside, peonidin-3-acetylglucoside, cyanidin-3-acetylglucoside and cyanidin-3-coumaroylglucoside were expressed as respective glucoside equivalents.

### 2.8 Determination of 4-EP and 4-EG by liquid-liquid extraction and GC–MS analysis

The extractions were carried out following and adapting the methodology described by [25].

### 2.9 Headspace wine aroma composition by solid phase microextraction (HS-SPME)

For the determination of the headspace aroma composition of red wines a validated method, confirmed in our laboratory was used [6].

### 2.10 Sensory evaluation

Sensory analysis was performed by a panel composed by six experts [26]. Fifteen attributes were selected: visual (limpidity, hue, colour intensity and oxidised), aroma (fruity, floral, vegetable character, phenolic and oxidised aroma) and taste and tactile/textural descriptors (taste–bitterness, acidity, tactile/textural–astringency, body, balance and persistence) using an adapted tasting sheet based on that recommended by the OIV [27]. The attributes were quantified using a five-point intensity scale [28]. Scales were anchored with the terms “low intensity” for score one and “high intensity” for score five, and panellists only scored integer values. All evaluations were conducted from 10:00 to 12:00 p.m. in an individual booth [29], using the recommended glassware according to [29]. A wine volume of 50 mL was used in order to be possible for the tasters to taste twice 25 mL of wine [30] and presented in random order [26].

### 2.11 Statistical treatment

Statistically significant differences between means were determined by analysis of variance (ANOVA) followed by Tukey honestly significant difference (HSD, 5% level) post-hoc test for the physicochemical data and a post-hoc Duncan test for sensory data. A principal component analyses was also performed to analyse the data and to study the relations between physicochemical ACs characteristics and wine volatile phenols removal and on phenolic and aromatic wine composition. These analyses were performed using Statistica 7 Software (StatSoft, Tulsa, OK U.S.A.). Generalised Procrustes Analysis [5] (GPA, XLSTAT-MX) of the sensory data was performed using XLSTAT (Addinsoft, Anglesey, UK). Multiple Factor Analysis (MFA, XLSTAT-RIB) of the sensory and chemical data were performed using XLSTAT (Addinsoft, Anglesey, UK).

## Figures and Tables

**Table 1:** Metal composition of activated carbons ashes.

Samples	Calcium	Iron	Magnesium	Potassium	Sodium	Copper	Aluminium
	(mg/g)	(mg/g)	(mg/g)	(mg/g)	(mg/g)	(µg/g)	(µg/g)
C1	1.64±0.11^b^	0.21±0.01^c^	1.64±0.09^d^	1.22±006^b^	152±0.07^e^	n.d.	n.d.
C2	4.78±0.09^e^	1.68±0.01^f^	3.12±0.03^e^	0.82±0.04^a^	1.03±0.08^d^	n.d.	n.d.
C3	6.02±0.22^f^	0.04±0.01^a^	1.06±0.01^a^	0.72±0.05^a^	0.96±0.01^c^	0.66±0.66^b^	n.d.
C4	0.92±0.08^a^	0.60±0.02^e^	1.44±0.05^c^	4.96±0.13^e^	0.56±0.05^a^	0.70±0.70^bc^	n.d.
C5	2.94±0.04^c^	0.12±0.01^b^	1.25±0.01^b^	3.89±0.18^d^	0.51±0.04^a^	0.77±0.77^c^	n.d.
C6	4.08±0.05^d^	0.28±0.03^d^	1.77±0.01^d^	1.92±0.08^c^	0.79±0.07^b^	0.76±0.76^c^	857.97±8.46
C7	5.26±0.16^e^	1.28±0.01^ab^	1.18±0.02^ab^	0.58±0.01^a^	0.70±0.03^a,b^	n.d.	n.d.

Values are presented as mean±standard deviation. Means within a column followed by the same letter are not significantly different ANOVA and Tuckey post-hoc test (*p*<0.05); n.d. – not detected.

**Table 2:** Assignment of FTIR bands of activated carbons main functional groups [2], [3], [4].

Wavenumber (cm^−1^)	Vibration Assignment	Functional Group
~1731	C=O stretching	Carboxylic acids and carboxylic anhydrides and lactones
~1637	C=O stretching	Quinone and keto-enol groups
~1590	C=C stretching	Aromatic
~1466	O-H bend	Carboxyl-carbonate structures
~1122	C-O stretching	Ethers
~1054	C-O(H) stretching	Phenolic groups
~649	C-C stretching	

**Table 3:** Procrustes Analysis of Variance (PANOVA) [5] of the sensory aromatic, taste and tactile/textural attributes data of volatile phenols free (T0) and volatile phenols spiked (TF) red wine and after treatment with different activated carbons (C1 to C7).

Source	DF	SS	MS	F	P
Residuals after scaling	160	41.48	0.259		
Scaling	5	10.06	2.012	7.759	<0.0001
Residuals after rotation	165	51.54	0.312		
Rotation	275	82.76	0.301	1.161	0.150
Residuals after translation	440	134.31	0.305		
Translation	55	147.59	2.683	10.349	<0.0001
Corrected total	495	281.900	0.570		

DF – Degrees of freedom.

**Table 4:** Scaling factors of experts for each configuration after GPA [5] of the sensory aromatic, taste and tactile/textural attributes data of volatile phenols free (T0) and volatile phenols spiked (TF) red wine and after treatment with different activated carbons (C1 to C7).

Object	Factor
Expert 1	0.8448
Expert 2	0.7695
Expert 3	0.9335
Expert 4	1.0430
Expert 5	1.2248
Expert 6	1.8165

**Table 5:** Headspace aroma profile of red wines before (volatile phenols free T0 and volatile phenols spiked TF) and after treatment with activated carbons with different physicochemical characteristics (C1–C7).

**Compounds**	**ID**^**$**^	**RI calculated**	RI^*^	**MW (g/mol)**	**Odour descriptor**	**ODT (mg/L)**	**T0**	**TF**	**C1**	**C2**	**C3**	**C4**	**C5**	**C6**	**C7**
Ethyl acetate	…	728	715	88.11	Fruity, sweet	7.5	23.9±1.5^b^	23.6±1.2^b^	30.6±2.1^c^	28.1±1.1^c^	24.2±0.6^b^	17.9±1.3^a^	19.1±0.6^a^	18.5±1.1^a^	17.0±0.8^a^
2-Methylpropan-1-ol	…	1118.5	1114	74.12	Bitter,green, harsh	0.2	1.80±0.05^c^	1.72±0.07^c^	1.98±0.16^cd^	2.01±0.16^d^	1.05±0.08^a^	1.39±0.11^b^	1.76±0.02^cd^	0.96±0.08^a^	0.93±0.04^a^
3-Methylbutan-1-ol acetate	std	1200	1126	130.18	Banana	0.03	6.89±1.04^e^	6.32±1.24^e^	3.40±0.47^c^	2.08±0.10^b^	0.82±0.05^a^	2.99±0.17^c^	0.36±0.06^a^	0.55±0.05^a^	3.98±0.67^d^
3-Methylbutan-1-ol	std	1223.3	1223	88.15	Alcohol, floral	30.0	190±3^f^	190±2^f^	166±6^e^	160±8^de^	149±2^d^	133±9^bc^	134±3^c^	119±6^ab^	111±4^a^
Ethyl hexanoate	std	1235.1	1238	144.21	Green apple, anise	0.014	24.3±3.8^c^	23.3±3.2^c^	5.39±0.06^b^	2.26±0.23^ab^	0.50±0.07^a^	0.17±0.01^a^	0.28±0.03^a^	0.40±0.04^a^	0.15±0.01^a^
Ethyl octanoate	std	1436.2	1436	172.27	Sweet, fruity, fresh	0.005	160±14^b^	156±12^b^	3.59±0.87^a^	1.79±0.38^a^	1.77±0.42^a^	n.d	n.d	n.d	n.d
Ethyl decanoate	std	1638.1	1646	200.32	Flowery, fruity	1.5	162±9^b^	164±7.^b^	3.64±0.66^a^	2.95±0.43^a^	2.97±0.19^a^	1.20±0.12^a^	n.d	n.d	n.d
Diethyl succinate	std	1682	1698	174.19	Light fruity	7.5	71.1±8.7^c^	76.0±10.4^c^	29.4±6.6^b^	17.8±2.5^ab^	15.2±0.9^a^	7.80±0.91^a^	9.29±0.80^a^	8.85±0.69^a^	7.68±0.61^a^
Phenylethyl acetate	std	1809.9	1833	164.2	Roses, flowery	0.25	4.37±1.17^b^	4.53±0.81^b^	0.69±0.11^a^	1.19±0.18^a^	0.42±0.06^a^	0.73±0.09^a^	0.48±0.08^a^	0.90±0.02^a^	0.55±0.03^a^
Hexanoic acid	std	1841.7	1857	116.16	Fatty acid, cheese	0.42	6.86±0.45^b^	6.69±0.29^b^	4.74±0.42^a^	6.36±0.64^b^	6.16±0.60^b^	5.88±0.50^ab^	5.85±0.40^ab^	5.60±0.42^ab^	4.80±0.25^a^
2-Phenylethanol	std	1912.7	1911	122.16	Roses, sweet	14.0	734±49^c^	710±55^c^	272±33^b^	299±45^b^	279±35^b^	181±21^a^	264±32^b^	278±35^b^	229±49^b^
4-Ethylguaiacol	std	1987	1989	152.18	Smoke	0.15	n.d.	57.4±8.5^b^	4.59±0.79^a^	11.7±1.6^a^	6.15±0.79^a^	5.36±0.35^a^	4.92±0.41^a^	6.39±0.70^a^	5.19±0.59^a^
Octanoic acid	std	2031.6	2030	144.21	Fatty acid, rancid	0.5	11.7±3.0^a^	11.4±0.6^a^	n.d.	n.d.	n.d.	n.d.	n.d.	n.d.	n.d.
4-Ethylphenol	std	2084	2142	122.16	Musty, spicy, phenolic	0.4	n.d.	4.09±0.89^b^	0.48±0.01^a^	0.77±0.05^a^	0.72±0.03^a^	0.59±0.06^a^	0.60±0.05^a^	0.81±0.05^a^	0.79±0.05^a^
Decanoic acid	…	2143	2196	172.27	Fatty, rancid, soap	1.0	10.9±3.2^b^	9.30±3.60^b^	n.d.	n.d.	n.d.	n.d.	n.d.	n.d.	n.d.
Dodecanoic acid	std	2254	2156	200.32	Fatty acid, soapy, waxy	6.1	3.46±0.48^c^	3.52±0.10^c^	n.d.	n.d.	n.d.	0.99±0.14^a^	1.33±0.19^b^	1.51±0.19^b^	1.38±0.14^b^.
						Total area	1412	1447	526.0	535.6	487.4	358.7	442.1	441.4	382.3
						% Reduction	…	…	63.6	63.0	66.3	75.2	69.5	69.5	73.6

Results expressed in absolute area (area*10^5^). Values are presented as mean±standard deviation; $ ID – Identification; std – Standard; * RI (retention index) from: [6], [7], [8]. MW (molecular weight). ODT (olfactory detection threshold). Odour descriptor from: [9], [10], [11]. Means within a column followed by the same letter are not significantly different ANOVA and Tuckey post-hoc test (*p*<0.05). n.d., not detected; volatile phenols free (T0) and volatile phenols spiked (TF) red wines and wines treated with seven activated carbons, C1 to C7.

**Table 6:** Molecular weight (MW), Log of octanol:water partition coefficient (LogP), polarizability and McGowan characteristic volumes of the headspace aroma compounds.

Compounds	MW (g/mol)	Log P	Polarizability	McGowan Characteristic Volume
Ethyl acetate	88.11	0.73	9.28	74.66
2-Methylpropan-1-ol	74.12	0.76	9.07	73.09
3-Methylbutan-1-ol acetate	130.18	2.25	15.20	116.93
3-Methylbutan-1-ol	88.15	1.16	11.03	87.18
Ethyl hexanoate	144.21	2.92	17.32	131.02
Ethyl octanoate	172.27	3.20	21.50	142.00
Ethyl decanoate	200.32	4.09	25.70	165.88
Diethyl succinate	174.19	1.26	18.38	138.46
Phenylethyl acetate	164.20	2.30	17.90	135.44
Hexanoic acid	116.16	1.81	13.27	102.84
2-Phenylethanol	122.16	1.36	13.87	105.69
4-Ethylguaiacol	152.18	2.47	16.75	125.65
Octanoic acid	144.21	3.05	23.57	131.02
4-Ethylphenol	122.16	2.58	13.86	105.69
Decanoic acid	172.26	4.09	21.61	159.20
Dodecanoic acid	200.32	4.20	25.85	187.38

Log P: ethyl acetate, 2-methylpropan-1-ol, 3-methylbutan-1-ol, phenylethyl acetate, 2-phenylethanol, octanoic acid, 4-ethylphenol, decanoic acid [12], dodecanoic acid [13], 3-methylbutan-1-ol acetate [14], ethyl hexanoate, diethyl succinate [15], ethyl octanoate, ethyl decanoate, hexanoic acid, 4-ethylguaiacol [16], polarizability [16]. McGowan characteristic volumes were determined according to [17].

**Table 7:** Total phenols, flavonoid phenols, non-flavonoid phenols, total anthocyanins and chromatic properties of red wines spiked with volatile phenols (TF) and after treatment with activated carbons with different physicochemical characteristics (C1–C7).

**Samples**	**Total phenols**	**Flavonoid phenols**	**Non-flavonoid phenols**	**Total anthocyanins**	**Colour intensity**	**Hue**	L*	a*	b*	ΔE*
	**(mg/L gallic acid)**	**(mg/L gallic acid)**	**(mg/L gallic acid)**	**(mg/L)**	**A.U.**					
TF	2023±2^d^	1623±14^c^	416±23^c^	354±5.6^c^	9.5±0.23^d^	0.71±0.01^a^	11.9±0.5^a^	42.47±0.66^a^	38.53±0.18^a^	–
C1	1808±0^b^	1493±14^a^	315±14^a^	337±3.7^b^	9.0±0.15^c^	0.72±0.02^a^	12.3±0.0^a^	42.83±0.09^a^	38.88±0.18^a^	0.74±0.53^a^
C2	1870±7^c^	1510±24^b^	360±16^b^	324±11.8^b^	8.8±0.23^c^	0.70±0.01^a^	12.6±0.0^a^	43.18±0.14^a^	38.67±0.23^a^	1.10±0.94^b^
C3	1745±19^a^	1413±33^a^	332±14^a^	281±0.0^a^	7.3±0.08^a^	0.73±0.00^a^	16.6±0.3^c^	47.33±0.35^d^	38.78±0.04^a^	6.78±1.25^d^
C4	1858±9^c^	1537±09^b^	322±00^a^	346±10.5^c^	9.4±0.36^d^	0.68±0.02^a^	11.7±0.5^a^	42.16±071^a^	38.48±0.70^a^	0.49±0.07^a^
C5	1817±7^c^	1505±07^b^	312±14^a^	310±14.2ª	8.3±0.02^b,c^	0.70±0.01^a^	13.9±02^b^	44.62±0.31^b^	38.93±0.30^a^	3.02±1.22^c^
C6	1825±14^c^	1487±19^a^	338±05^a^	311±2.5^a^	8.1±0.18^b^	0.71±0.01^a^	14.7±0.1^b^	45.59±0.08^c^	39.51±0.16^a^	4.32±0.91^c^
C7	1767±16^a^	1448±16^a^	318±00^a^	288±4.9^a^	7.3±0.11^a^	0.73±0.00^a^	16.4±0.2^c^	47.24±0.24^d^	39.16±0.04^a^	6.51±0.67^d^

Values are presented as mean±standard deviation; Means within a column followed by the same letter are not significantly different ANOVA and Tuckey post-hoc test (*p*<0.05). L*(%) – lightness, a* - redness, b* - yellowness, ΔE* – colour difference. The values corresponding to ΔE* were obtained taking as a reference the untreated wine (TF). A.U. – absorbance units, wines treated with seven activated carbons, C1 to C7.

**Table 8:** Phenolic acids (mg/L) of red wines spiked with volatile phenols (TF) and after treatment with activated carbons with different physicochemical characteristics (C1–C7).

**Samples**	**Gallic acid**	**Catechin**	***trans-*****Caftaric acid**	**GRP**	**Coutaric acid**	**Caffeic acid**	**Coumaric acid**	**Ferulic acid**	**Caffeic acid ethyl ester**	**Coumaric acid ethyl ester**
TF	9.92±1.03^a^	13.33±0.94^a^	31.70±0.27^b^	0.11±0.00^a^	12.14±0.04^c^	3.17±0.19^c^	3.96±1.56^b^	0.79±0.06^b^	1.06±0.25^b^	2.89±0.03^d^
C1	5.69±0.35^a^	7.49±3.76^b^	27.91±0.87^a^	0.20±0.06^a^	9.72±0.07^a^	0.66±0.09^a^	0.62±0.13^a^	0.12±0.01^a^	0.10±0.01^a^	2.28±0.01^d^
C2	6.28±2.30^a^	13.85±0.05^a^	29.95±0.70^a^	0.14±0.04^a^	11.22±0.11^b,c^	1.71±0.10^b^	1.23±0.09^a^	0.12±0.01^a^	0.16±0.04^a^	1.90±0.07^c^
C3	6.28±2.30^a^	12.29±0.05^a^	29.64±0.13^a^	0.25±0.11^a^	10.79±0.13^b^	1.11±0.09^a^	0.84±0.06^a^	0.05±0.01^a^	0.03±0.02^a^	0.93±0.01^a^
C4	6.28±2.30^a^	12.24±0.21^a^	29.68±0.21^a^	0.30±0.05^a^	10.56±0.10^b^	1.00±0.01^a^	0.46±0.49^a^	0.06±0.01^a^	0.09±0.05^a^	2.60±0.02^d^
C5	6.28±2.31^a^	11.88±0.21^a^	29.64±0.40^a^	0.37±0.25^a^	10.51±0.09^b^	0.84±0.05^a^	0.27±0.13^a^	0.79±0.04^b^	0.03±0.01^a^	1.50±0.07^b^
C6	6.28±2.31^a^	13.09±0.08^a^	30.83±0.49^a^	0.48±0.14^a^	10.98±0.10^b^	0.93±0.02^a^	0.10±0.01^a^	0.73±0.07^b^	0.09±0.05^a^	1.45±0.01^b^
C7	6.28±2.31^a^	11.76±0.10^a^	29.67±0.18^a^	0.06±0.09^a^	10.23±0.69^b^	0.75±0.14^a^	0.07±0.02^a^	0.67±0.06^b^	0.02±0.00^a^	1.29±0.42^b^

Values are presented as mean ± standard deviation; Means within a column followed by the same letter are not significantly different ANOVA and Tuckey post-hoc test (*p*<0.05). GRP - 2-*S*-glutathionyl caftaric acid.

**Table 9:** Monomeric anthocyanin composition (mg/L) of red wines spiked with volatile phenols (TF) and after treatment with activated carbons with different physicochemical characteristics (C1–C7).

**Samples**	**Del-3-Glc**	**Cya-3-Glc**	**Pet-3-Glc**	**Peo-3-Glc**	**Mal-3-Glc**	**Del-3-AcGlc**	**Cya-3-AcGlc**	**Pet-3-AcGlc**	**Peo-3-AcGlc**	**Mal-3-AcGlc**	**Del-3-CoGlc**	**Cya-3-CoGlc**	**Pet-3-CoGlc**	**Peo-3-CoGlc**	**Mal-3-CoGlc**
TF	1.00±0.21^a^	5.94±0.07^c^	10.64±0.11^e^	11.51±0.11^b^	59.28±0.79^d^	2.67±0.35^c^	n.d.	n.d.	0.11±0.01^a^	7.51±0.15^c^	n.d.	0.06±0.04^a^	n.d.	0.71±0.06^a^	9.02±0.08^c^
C1	0.83±0.14^a^	5.23±0.33^ab^	9.15±0.29^d^	9.22±0.58^b^	52.48±0.02^b^	2.14±0.08^b^	n.d.	n.d.	n.d.	6.64±0.03^b^	n.d.	n.d.	n.d.	n.d.	7.18±0.29^c^
C2	0.95±0.07^a^	5.53±0.90^bc^	8.95±0.25^c^	9.20±0.74^b^	51.16±0.84^b^	1.85±0.06^b^	n.d.	n.d.	n.d.	6.08±0.28^b^	n.d.	n.d.	n.d.	n.d.	5.86±0.48^b^
C3	0.97±0.05^a^	4.32±0.31^ab^	7.67±0.05^a^	8.65±0.18^b^	43.99±0.30^a^	0.97±0.12^a^	n.d.	n.d.	n.d.	4.32±0.18^a^	n.d.	n.d.	n.d.	n.d.	2.48±0.16^a^
C4	0.97±0.14^a^	5.57±0.44^bc^	9.43±0.20^d^	9.75±0.89^b^	55.39±2.49^c^	2.14±0.11^b^	n.d.	n.d.	n.d.	7.83±0.91^d^	n.d.	n.d.	n.d.	n.d.	8.77±1.27^c^
C5	0.65±0.07^a^	4.47±0.01^ab^	8.12±0.09^b^	7.97±0.70^a^	49.00±0.99^b^	1.43±0.04^ab^	n.d.	n.d.	n.d.	5.53±0.27^a^	n.d.	n.d.	n.d.	n.d.	4.16±0.06^a^
C6	0.61±0.08^a^	4.22±0.19^ab^	7.73±0.32^a^	8.27±0.04^a^	48.31±0.19^b^	1.00±0.44^a^	n.d.	n.d.	n.d.	5.71±0.39^a^	n.d.	n.d.	n.d.	n.d.	4.04±0.19^a^
C7	0.92±0.15^a^	3.68±0.23^a^	7.04±0.43^a^	7.44±1.42^a^	43.57±1.21^a^	0.91±0.10^a^	n.d.	n.d.	n.d.	4.18±0.24^a^	n.d.	n.d.	n.d.	n.d.	2.64±0.08^a^

Values are presented as mean±standard deviation; Del-3-Glc-Delphinidin-3-glucoside, Cya-3-Glc-Cyanidin-3-glucoside, Pet-3-Glc-Petunidin-3-glucoside, Peo-3-Glc-Peonidin-3-glucoside, Mal-3-Glc-Malvidin-3-glucoside, Del-3-AcGlc-Delphinidin-3-acetylglucoside, Cya-3-AcGlc-Cyanidin-3-acetylglucoside, Pet-3-AcGlc-Petunidin-3-acetylglucoside, Peo-3-AcGlc-Peonidin-3-acetylglucoside, Mal-3-AcGlc-Malvidin-3-acetylglucoside, Del-3-CoGlc-Delphidin-3-coumaroylglucoside, Cya-3-CoGlc-Cyanidin-3-coumaroylglucoside, Pet-3-CoGlc-Petunidin-3-coumaroylglucoside, Peo-3-CoGlc-Peonidin-3-coumaroylglucoside; Mal-3-CoGlc-Malvidin-3- coumaroylglucoside. Means within a column followed by the same letter are not significantly different ANOVA and Tuckey post-hoc test (*p*˂0.05).
